# Supplementary figures and images for: Trans‐ethnic polygenic risk scores for body mass index: An international hundred K+ cohorts consortium study
Source: Clin Transl Med. 2023 Jun 19;13(6):e1291. doi: 10.1002/ctm2.1291 (PMC10280047; doi:10.1002/ctm2.1291)

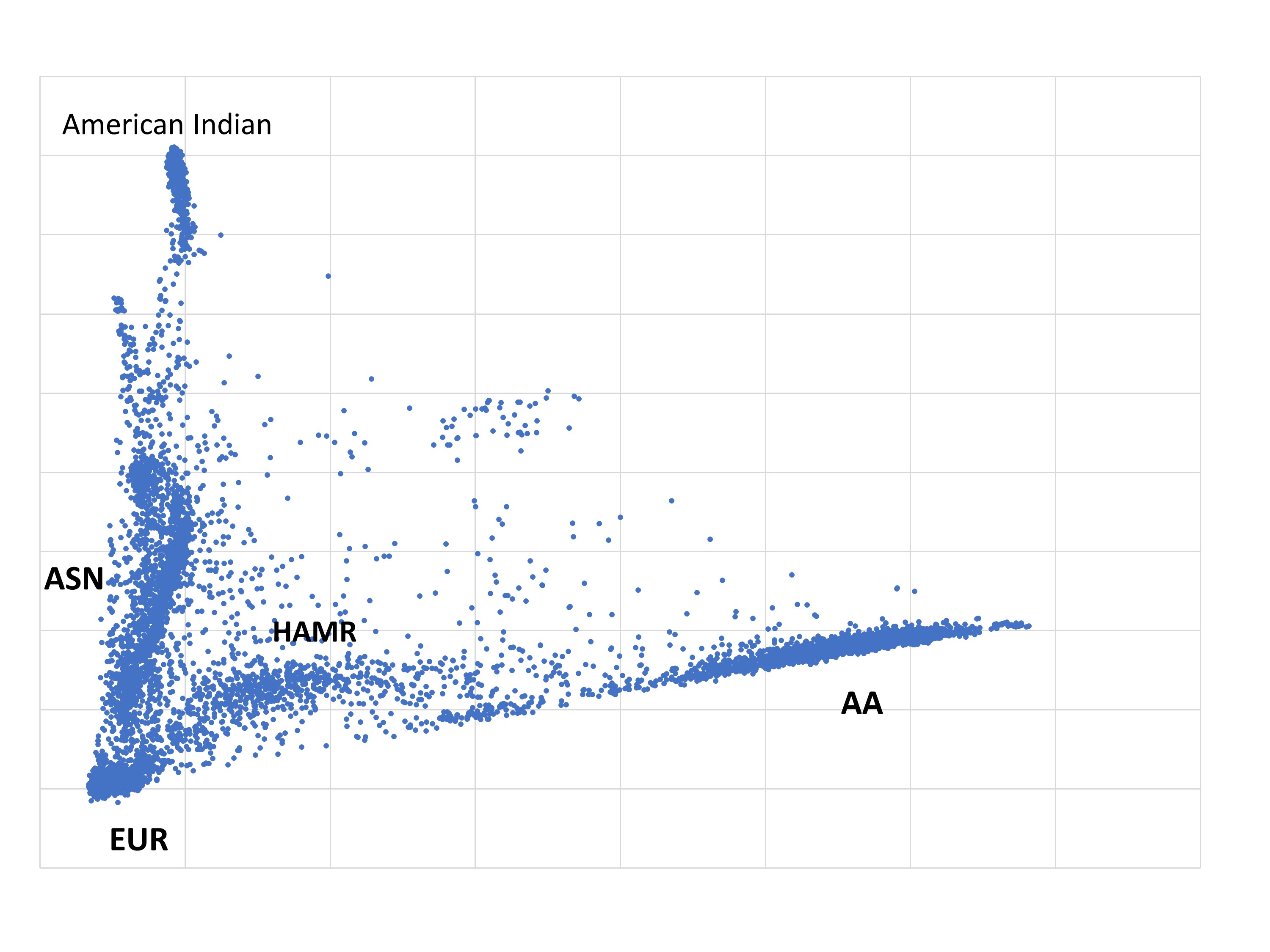

Supplement: Supplementary file 2 — Supporting Information [file CTM2-13-e1291-s003.jpg]
