# Supplementary material for: Trans‐ethnic polygenic risk scores for body mass index: An international hundred K+ cohorts consortium study
Source: Clin Transl Med. 2023 Jun 19;13(6):e1291. doi: 10.1002/ctm2.1291 (PMC10280047; doi:10.1002/ctm2.1291)
Supplement: Supplementary file 3 — Supporting Information [file CTM2-13-e1291-s001.docx]

**Funding information**

The study was supported by:

All authors are supported by the International HundredK+ Cohorts Consortium (IHCC), which has been created in collaboration with the Global Alliance for Genomics and Health (GA4GH) and the Global Genomics Medicine Collaborative (G2MC) with support from the National Institute of Health and the Wellcome Trust.

CHOP funding: Institutional Development Funds from the Children’s Hospital of Philadelphia to the Center for Applied Genomics, and The Children’s Hospital of Philadelphia Endowed Chair in Genomic Research.

The Shanghai Women's Health Study (US NIH, R37CA070867, UM1CA182910), and the Shanghai Men's Health Study (US NIH, R01CA082729, UM1CA173640), are funded by the National Institutes of Health.

ELSA-Brasil is funded by the Brazilian Ministry of Health (Department of Science and Technology) and the Ministry of Science, Technology and Innovation (FINEP, Financiadora de Estudos e Projetos), and CNPq (the National Council for Scientific and Technological Development).

The Norwegian Mother, Father and Child Cohort Study (MoBa) is supported by the Norwegian Ministry of Health and Care Services and the Ministry of Education and Research. We are grateful to all the participating families in Norway who take part in this on-going cohort study. We thank the Norwegian Institute of Public Health (NIPH) for generating high-quality genomic data. This research is part of the HARVEST collaboration, supported by the Research Council of Norway (#229624). We also thank the NORMENT Centre for providing genotype data, funded by the Research Council of Norway (#223273), South East Norway Health Authorities and Stiftelsen Kristian Gerhard Jebsen. We further thank the Center for Diabetes Research, the University of Bergen for providing genotype data and performing quality control and imputation of the data funded by the ERC AdG project SELECTionPREDISPOSED, Stiftelsen Kristian Gerhard Jebsen, Trond Mohn Foundation, the Research Council of Norway, the Novo Nordisk Foundation, the University of Bergen, and the Western Norway Health Authorities.

Participants in the INTERVAL randomized controlled trial were recruited with the active collaboration of NHS Blood and Transplant (www.nhsbt.nhs.uk), which has supported fieldwork and other elements of the trial. DNA extraction and genotyping were co-funded by the National Institute for Health Research (NIHR), the NIHR BioResource (http://bioresource.nihr.ac.uk) and the NIHR Cambridge Biomedical Research Centre (BRC) (no. BRC-1215-20014). The academic coordinating centre for INTERVAL was supported by core funding from the NIHR Blood and Transplant Research Unit in Donor Health and Genomics (no. NIHR BTRU-2014-10024), UK Medical Research Council (MRC) (no. MR/L003120/1), British Heart Foundation (nos SP/09/002, RG/13/13/30194 and RG/18/13/33946) and the NIHR Cambridge BRC (no. BRC-1215-20014). A complete list of the investigators and contributors to the INTERVAL trial is provided in ref.17. The academic coordinating centre thanks blood donor centre staff and blood donors for participating in the INTERVAL trial. This work was supported by Health Data Research UK, which is funded by the UK MRC, Engineering and Physical Sciences Research Council (EPSRC), Economic and Social Research Council, Department of Health and Social Care (England), Chief Scientist Office of the Scottish Government Health and Social Care Directorates, Health and Social Care Research and Development Division (Welsh Government), Public Health Agency (Northern Ireland), British Heart Foundation and Wellcome. The views expressed in this manuscript are those of the authors and not necessarily those of the NIHR or the Department of Health and Social Care.
